# Editorial Research Topic “Chemokines and chemokine receptors in brain homeostasis”

**DOI:** 10.3389/fncel.2015.00132

**Published:** 2015-04-08

**Authors:** Richard M. Ransohoff, Flavia Trettel

**Affiliations:** ^1^BiogenCambridge, MA, USA; ^2^Department of Physiology and Pharmacology, Istituto Pasteur Fondazione Cenci Bolognetti, University of Rome “Sapienza,”Rome, Italy

**Keywords:** Chemokines, brain homeostasis, brain pathology, pain, inflammation, neuro-glia cross-talk, glioma stem cell, neurotransmission

The present *Frontiers* eBook “Chemokines and chemokine receptors in brain homeostasis” grew from a delightful conference held in Rome, Italy from 25th to 27th, 2013. It's our hope that this eBook will enable you to sense the conviviality and intellectual ferment of that weekend, as you won't be able to taste the wine or pasta.

The 11 articles in this compilation (Biber and Boddeke, 2014; Clark and Malcangio, 2014; Freitag and Miller, 2014; Guyon, 2014; Hosking and Lane, 2014; Limatola and Ransohoff, 2014; Michlmayr and Lim, 2014; Mony et al., 2014; Rosito et al., 2014; Williams et al., 2014; Würth et al., 2014) comprise a spectrum of chemokine neurobiology much of which will be unfamiliar (and thus, one hopes, fascinating) both to chemokine aficionados and neuroscientists. Only one paper (Mony et al., 2014) addresses purely the best-known aspect of chemokine action in the context of neurological pathology: their role in accumulation of inflammatory blood-derived leukocytes in the central nervous system (CNS). Williams et al. (2014) also study leukocyte recruitment to the CNS but additionally evaluate evidence that CXCL12 (the chemokine on which they focus) can either promote or degrade neural function during altered homeostasis. Limatola and Ransohoff (2014) examine how a neuronal chemokine (CX3CL1) signals to its microglial receptor (CX3CR1) to help determine cell death or survival in the context of varied pathological processes. One group of scientists (Rosito et al., 2014) present their data about how chemokine-mediated cell-cell communication among neurons and glia supports neuronal function after focal cerebral ischemia. Two groups (Hosking and Lane, 2014; Michlmayr and Lim, 2014) integrate these topics (chemokine-regulation of inflammatory host defense; chemokine effects on cell death or survival) by utilizing informative models of encephalitis. Three groups (Biber and Boddeke, 2014; Clark and Malcangio, 2014; Freitag and Miller, 2014) describe their work using chemokine biology to unravel the puzzle of neuropathic pain. There is a heterogeneity of additional topics. Guyon (2014) examines how CXCL12 signaling modulates GABA neurotransmission. Würth et al. (2014) study the same chemokine (CXCL12) now in the guise of an autocrine and paracrine signal to promote growth of glioma stem cells and maintain a supportive microenvironment.

It will be appreciated that the common rubric “Chemokines are chemotactic cytokines” no longer encompasses even a tiny fraction of the activities of these versatile mediators in CNS physiology and pathology. The predominant focus currently lies on CXCL12 and CX3CL1 but other players (ELR+ CXC chemokines; CCL21; CXCL16) also begin to be heard from. Given the pace at which molecular components of development and disease are being identified, it is plausible to hope that this eBook represents only the tip of an iceberg which will calve rapidly into knowledge that promotes the treatment of neurological disorders.

## Conflict of interest statement

The authors declare that the research was conducted in the absence of any commercial or financial relationships that could be construed as a potential conflict of interest.
